# Motor skill learning modulates striatal extracellular vesicles’ content in a mouse model of Huntington’s disease

**DOI:** 10.1186/s12964-024-01693-9

**Published:** 2024-06-11

**Authors:** Júlia Solana-Balaguer, Pol Garcia-Segura, Genís Campoy-Campos, Almudena Chicote-González, Joaquín Fernández-Irigoyen, Enrique Santamaría, Esther Pérez-Navarro, Mercè Masana, Jordi Alberch, Cristina Malagelada

**Affiliations:** 1 Departament de Biomedicina, Institut de Neurociències, Facultat de Medicina i Ciències de la Salut, Universitat de Barcelona, Casanova 143, North Wing, 3rd Floor, Barcelona, Catalonia, 08036 Spain; 2 Centro de Investigación Biomédica en Red sobre Enfermedades Neurodegenerativas (CIBERNED), Barcelona, Spain; 3 Proteored-ISCIII, Proteomics Unit, Departamento de Salud, UPNA, Navarrabiomed, Pamplona, IdiSNA Spain; 4 Institut d’Investigacions Biomèdiques August Pi i Sunyer (IDIBAPS), Barcelona, Spain

**Keywords:** Extracellular vesicles, Motor learning, Huntington’s disease, Cortico-striatal activation, Striatum, Proteomics

## Abstract

Huntington’s disease (HD) is a neurological disorder caused by a CAG expansion in the Huntingtin gene (*HTT*). HD pathology mostly affects striatal medium-sized spiny neurons and results in an altered cortico-striatal function. Recent studies report that motor skill learning, and cortico-striatal stimulation attenuate the neuropathology in HD, resulting in an amelioration of some motor and cognitive functions. During physical training, extracellular vesicles (EVs) are released in many tissues, including the brain, as a potential means for inter-tissue communication. To investigate how motor skill learning, involving acute physical training, modulates EVs crosstalk between cells in the striatum, we trained wild-type (WT) and R6/1 mice, the latter with motor and cognitive deficits, on the accelerating rotarod test, and we isolated their striatal EVs. EVs from R6/1 mice presented alterations in the small exosome population when compared to WT. Proteomic analyses revealed that striatal R6/1 EVs recapitulated signaling and energy deficiencies present in HD. Motor skill learning in R6/1 mice restored the amount of EVs and their protein content in comparison to naïve R6/1 mice. Furthermore, motor skill learning modulated crucial pathways in metabolism and neurodegeneration. All these data provide new insights into the pathogenesis of HD and put striatal EVs in the spotlight to understand the signaling and metabolic alterations in neurodegenerative diseases. Moreover, our results suggest that motor learning is a crucial modulator of cell-to-cell communication in the striatum.

**Supplementary Information:**

The online version contains supplementary material available at 10.1186/s12964-024-01693-9.

## Introduction

Huntington’s disease (HD) is a neurodegenerative autosomal-dominant genetic disorder caused by an abnormal CAG (Cytosine-Adenine-Guanine) expansion in the huntingtin (*HTT)* gene. *HTT* gene codes for the huntingtin protein (htt), which in HD patients, presents an abnormal number of glutamine repeats (polyQ > 36). This mutation induces an aberrant aggregation and accumulation of the mutant htt (mhtt) [1] which causes specific vulnerability to medium-sized spiny neurons [2, 3] and impairs the synaptic connectivity between the cortex and striatum [4, 5]. This degeneration results in choreiform movements, cognitive deficits, and even psychiatric symptoms [6–8].

Current therapies for HD are directed to treat symptoms, as there are no disease-modifying strategies yet [9]. However, recent studies have stablished that environmental factors such as physical activity have a significant impact in the progression of the disease [10]. For example, in mouse models of HD, physical training seems to decrease protein aggregation, cell death and mitochondrial dysfunction. Moreover, physical training showed an improvement in motor function, cognition and slowed down disease progression in both HD mouse models and in patients. It is important to note that, in these studies, the physical activity periods greatly differ between studies, from 3 days to 5 months (reviewed in [11]). Motor skill learning tasks involve at least acute physical training, and these intertwined events activate the cortico-striatal synaptic pathway [12, 13]. Importantly, the activation of this circuitry seems to be beneficial on some symptomatology of HD [14].

However, the mechanisms behind the therapeutic effects of motor learning and cortico-striatal activation are not completely understood. Physical training has systemic consequences on the body, impacting most organs, including the brain. It has been shown that, along with several classical cytokines an myokines, extracellular vesicles (EVs) are released into the circulation during training as potential means for inter-tissue communication [15].

EVs are small membrane-bound vesicles released by cells that have been proven as versatile messengers since they contain biologically active proteins, RNAs and lipids [16–18]. Although several studies involve EVs in the propagation of toxic proteins [19–22], EVs have also been shown to be key players in ensuring the physiological functions in the brain, as they act as modulators of neurogenesis [23], synaptic plasticity [24] and myelination [25].

There are different types of EVs, distinguished by size and biogenesis. Among them, exosomes are ∼60 to 120 nm vesicles produced by the endosomal system and secreted by the fusion of multivesicular bodies with the plasma membrane. In contrast, microvesicles are bigger particles, between ∼100 nm and 1 μm released by outward budding from plasma membrane [26, 27].

EVs participate in training-mediated adaptation processes that involve signaling across tissues and organs [28]. However, to date, it is unknown how motor learning, and therefore the activation of cortico-striatal pathway, could affect the profile of EVs released in the striatum.

For this reason, we investigated the potential effect of motor learning in the modulation of the crosstalk between cells in the striatum via EVs and how this is impaired in a pathologic context. Here, we found that R6/1 striatal EVs presented a differential signature in size and protein content, confirming alterations in biological pathways already described to be affected in HD. Motor learning exposure, although insufficient to revert the overall HD phenotype, restored striatal R6/1 EVs concentration and protein deficiencies associated to metabolism and neurodegeneration.

## Materials and methods

### Animals

Heterozygous R6/1 transgenic mice, maintained in a B6CBA background, were used as a model of HD (RRID: IMSR_JAX:006471). WT littermate animals were used as the control group. R6/1 mice express exon 1 of human mhtt with 115 CAG repeats, which codes for part of the N-terminal regions of the protein, including the polyglutamine stretch. Transgene expression is driven by the human huntingtin promoter. Male animals of 8 weeks of age were used. All procedures were carried out in accordance with the National Institutes of Health Guide of the Care and Use of Laboratory Animals and approved by the local animal care committee of the Universitat de Barcelona (315/18 P10), following European (2010/63/UE) and Generalitat de Catalunya (10,141-P10) regulations.

Mice were housed under controlled conditions: 22ºC, 40–60% humidity in a 12 h light/dark cycle) and with water and food available ad libitum.

### Accelerating rotarod

2-month-old WT and R6/1 mice were subjected to the accelerating rotarod test. Mice were placed on a 3 cm rod with an increasing speed from 4 to 40 rpm over 5 min, as in Martín-Flores, N. et al. (2020) [29], with minor modifications. Latency to fall from the rod was recorded. Briefly, accelerating rotarod test was performed for 3 days, 4 trials per day. Trials 1 to 2 and trials 3 to 4 were separated by 15 min. Trials 2 to 3 were separated by 30 min to let the animals recover from the physical activity. Naïve animals’ group were presented to the rotarod the first day (they were placed on the rod) but they were not trained. 1 h and 30 min after the last trial, both naïve and trained animals were euthanized by cervical dislocation and both right and left striatum were dissected out and frozen at -80ºC until EVs isolation.

### Extracellular vesicles isolation from mice tissue

EVs were isolated from the striatal tissue as in Pérez-Gonzalez R. (2017) [30], with some modifications. Briefly, frozen striatum was weighted before starting the EVs isolation. Tissue was chopped and chemically digested for 15 min at 37 ºC with ~ 20 units of papain solution (Labclinics) in Hibernate-A medium (Thermo Fisher Scientific). The enzymatic reaction was stopped adding cold Hibernate-A supplemented with 1X PhosSTOP™ phosphatase inhibitors cocktail, 1X cOmplete™ protease inhibitors cocktail, 2mM PMSF, 5µM E-64 (all from Merk). Tissue was then homogenized and centrifuged at 300 *x g* for 10 min, to eliminate cell debris. Supernatant was sequentially filtered out in 0.45 μm filter and in 0.20 μm filter. Then a 2,000 *x g* centrifugation for 10 min was performed to remove apoptotic bodies (P2000) and a 10,000 *x g* centrifugation for 30 min to pellet large microvesicles (P10K). The supernatant was ultracentrifuged at 100,000 *x g* two times for 70 min, to pellet down the small EVs (sEVs). The pellet was resuspended in 1X PBS and applied to the size-exclusion chromatography (SEC) column.

SEC columns were prepared using puriflash columns dry load empty (Interchim), loaded with sepharose (GE Healthcare) in azide solution, as in Gámez-Valero, A. et al. (2016) [31]. The columns were washed in 1X PBS before use. The fraction containing sEVs was applied to the column and 35 fractions of 500 µL were collected. Protein concentration of each fraction was measured using the NanoDrop™ One Microvolume UV-VIS Spectrophotometer (Thermo Fisher Scientific) and vesicle size and concentration with the NanoSight NS300 equipment.

The fractions containing the peak of vesicles were pulled together and an ultracentrifugation of 100,000 *x g* for 70 min was performed to pellet the sEVs. All centrifugations were performed at 4 ºC. The pellet was resuspended in 1X PBS for NTA analysis and negative staining, in 1X RIPA buffer (Cell Signaling Technologies) for western blotting (WB) or in 1X lysis buffer (7 M urea, 2 M thiourea and 50 mM dithiothreitol) for proteomic analysis.

### Western blotting

The striatal tissue not used for EVs isolation was processed as in Pérez-Sisqués, L. (2022) [32] to obtain the homogenate, and protein concentration was measured using Bradford reagent (Bio-rad). P2000, P10K, and EVs fractions were resuspended in 1X RIPA buffer (supplemented with 1X PhosSTOP™ phosphatase inhibitors cocktail, 1X cOmplete™ protease inhibitors cocktail, 2mM PMSF and 5µM E-64) and protein concentration was measured using microBCA™ (Thermo Fisher Scientific).

The following primary antibodies were used (1:1,000 if not stated otherwise): mouse monoclonal anti-Alix (Thermo Fisher Scientific, #MA183977, 1:500) mouse monoclonal anti-TSG101 (Abcam, #ab83), mouse monoclonal anti-Flotillin-1 (BD Bioscience, #610,821), mouse monoclonal anti-TOMM20 (abcam, #ab56783), mouse monoclonal anti-phospho-p44/42-Thr202/Tyr204 MAPK (ERK1/2) (Cell Signaling Technology, #9106), rabbit polyclonal anti-ERK (Santa Cruz Biotechnologies, #sc-93), rabbit polyclonal anti-phospho-Akt-Ser473 (Cell Signaling Technology, #4060S), rabbit polyclonal anti-phospho-RPS6-Ser235/236 (Cell Signaling Technology, #4858S), rabbit polyclonal anti-Akt (Cell Signaling Technology, #4691S) and mouse monoclonal anti-RPS6 (Cell Signaling Technology, #2317).

The loading control was obtained by incubation with an anti-α-actin-Peroxidase antibody (1:100,000; Merck, #A3854) or with rabbit polyclonal anti-vinculin (Cell Signaling Technology, #4650). Horseradish peroxidase-conjugated goat anti-mouse and anti-rabbit secondary antibodies (1:10,000) were obtained from Thermo Fisher Scientific (1:10,000, #31,430 and #31,460, respectively).

In the case of gels containing both lysates and EVs samples, membranes were cut and lysates and EVs were incubated separately with the antibodies, to avoid signal sequestration.

Chemiluminescent images were acquired using a Chemidoc imager (BioRad) and quantified by computer-assisted densitometric analysis (ImageJ). All the blots used for the figures are shown in Figure S4.

### Transmission electron microscopy

For transmission electron microscopy (TEM), the EVs pellet was resuspended in 2% paraformaldehyde (PFA, Electron Microscopy Sciences) in 1X PBS and deposited on Formvar-carbon-coated 400-mesh copper grids for 25 min until adsorption. Grids were then transferred to a ~ 30 µL drop of 2% saturated aqueous uranyl acetate as a contrast agent. The excess mixture was removed by capillarity using filter paper and grids were washed in water. When dried, samples were observed under a JEOL JEM-1010 (100 kV) microscope (JEOL, Ltd.) and image acquisition was made with a Gatan Orius CCD Camera (AMETEK, Inc.) at 200,000x magnification.

### Nanoparticle tracking analysis

EVs size and concentration were analyzed by nanoparticle tracking analyses (NTA), using NanoSight NS300 equipment (Spectris). Samples were diluted in 1X Phosphate buffered-saline (PBS) and three videos of 60 s were recorded per sample. Videos were analyzed with the NTA Software (NTA v3.4 Build 3.4.4) to determine the size and concentration of particles in EVs samples. Settings: Camera sCMOS, Laser Blue466, Camera Level 12, Slider Shutter 1200, Slider Gain 146, Shutter/ms 30, Frame rate/fps 25, Syringe Pump Speed/AU 50, Detection Threshold 5, Total Frames analyzed 1498. EVs concentration was normalized to the weight of the tissue used for EVs isolation.

### Proteomics

Samples were processed and analyzed at the Proteomics Platform of Navarrabiomed-IdiSNA Center for Biomedical Research. For sample preparation, protein extracts were diluted in Laemmli sample buffer (4%) and were then loaded into a 0.75-mm-thick polyacrylamide gel containing a 4% stacking gel cast over 12.5% resolving gel. To concentrate the entire proteome at the stacking/resolving gel interface, the run was stopped as soon as the front entered 3 mm of the resolving gel. Gel was then stained using Coomassie Brilliant Blue and bands were excised and digested using 1:20 trypsin solution at 37ºC for 16 h as previously described [33]. Peptide fragments were purified and concentrated using C18 Zip Tip Solid Reverse Phase columns (Millipore). Samples were then separated by reverse phase LC-MS/MS using an UltiMate 3000 UHPLC System (ThermoFisher) fitted with a column in an acetonitrile gradient coupled to the Orbitrap Exploris 480 MS (ThermoFisher). Mass range was set to 375–1500 ppm. All the other acquisition parameters were set as previously described [34]. The MaxQuant computing platform v.1.6.17.0 [35]. and the environment-integrated Andromeda search engine [36] were used to process the raw files. For peptide identification, a target-decoy search strategy [37] was performed against a target/decoy version of the rat UniProt database without isoforms with a maximum peptide mass of 7500 Da. The false discovery rate limit was set to 1% on both the peptide and protein identification levels. The Perseus software v.1.6.14.0 [38] was used for statistical and differential expression analyses. Only proteins with at least two identified peptides were considered for further analyses. The option “two samples t-test” was used to compare experimental conditions. Here, comparisons were statistically different if the following conditions were met: (i) Benjamini-Hochberg adjusted p-values under 0.05 and a (ii) log2 fold-change over 0.3 and under − 0.3. R (v.4.2.1) packages ComplexHeatmap [39], EnhancedVolcano and mixOmics [40], were used for multivariate data analysis and visualization.

To proceed with dimensionality reduction in the proteomic analyses, partial least square discriminant analysis (PLS-DA) was first used. The variables that contribute to a better separation of the classes were selected in each projection, using the variable importance projection metric (VIP). The variables with a VIP score > 1.5 were selected and principal component analysis (PCA) was performed as implemented in mixOmics R package [40]. All the proteomic and dimensionality reduction analyses were performed using the mixOmics R package. For the 2D and 3D representation, ggplot2 [41], and rgl [42] R packages were used, respectively.

Gene site enrichment analysis (GSEA) of the differential protein sets in the different experimental groups was computed using R package gProfileR (v. 0.7.0) [43]. The differential proteins with FDR < 5% with positive and negative fold change in the same analysis were tested. The background was set to the input set of proteins detected by mass spectrometry. External gene names of the differential proteins were used as a query. Organism was set as a mouse. Electronic annotations were excluded, the p-value correction method was set to “fdr” and results with FDR < 5% were considered. igraph (v.1.5.0) [44] and networkD3 (v.0.4) [45] R packages were used for network representation of the results. ggplot2 version R package was used for other statistical results representation, such as the UpSet plot [41].

### Statistics

All experiments were performed with 4 animals per group (*n* = 4) and data was reported as mean ± SEM. Normal distribution was considered when all the data passed one of the following normality tests: D’Agostino-Pearson, Shapiro-Wilk, and Kolmogorov-Smirnov. Two-way ANOVA with Bonferroni’s post hoc test was used to compare multiple groups. Values of *P* < 0.05 were considered statistically significant.

## Results

### Isolation and characterization of EVs derived from R6/1 mouse striatum

**Fig. 1:**
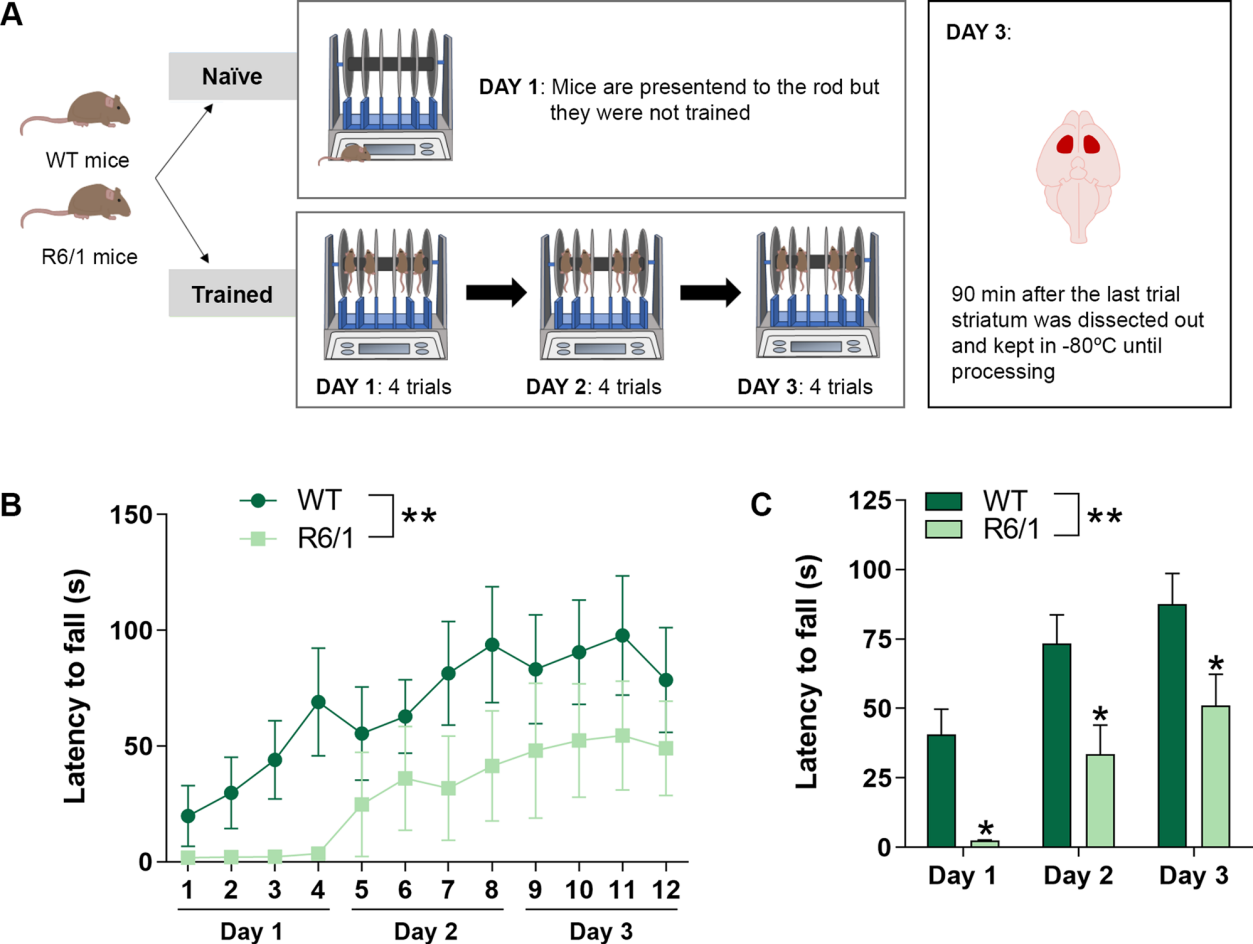
Accelerating rotarod training in WT and R6/1 mice. **(A)** Schematic representation of the experimental procedure. 2-month-old WT and R6/1 transgenic mice were divided in two groups: the naïve group was presented the first day to the rod, but no training was performed, and the trained group was physically trained for 3 consecutive days, with 4 trials per day. 90 min after the last trial, the striata was dissected out and kept at -80ºC until processing for EVs isolation. **(B)** Latency to fall at accelerating speeds (4–40 rpm) over 5 min. **(C)** Latency to fall. Data is represented as the mean of the 4 trials per day. Values are represented as mean ± SEM (*n* = 4). Data were analyzed by two-way ANOVA followed by Bonferroni’s post hoc test. (*P < 0.05, vs. WT)

To investigate the potential effect of the cortico-striatal pathway activation, via motor skill learning, on striatal EVs profile, we subjected WT and R6/1 mice to the accelerating rotarod test, for 3 consecutive days. Half of the animals, grouped as naïve, were presented to the rod the first day but no training was performed (Fig. 1A).

Only four animals per group were sufficient to significantly reproduce the disease-associated deficits in the rotarod task, as expected, in line with our own previous work. We observed that both WT and R6/1 mice improved their performance per day, confirming they were properly trained, but the R6/1 mice had motor learning deficits, compared to WT, since the latency to fall was shorter, as previously described [29] (Fig. 1B & C).

**Fig. 2:**
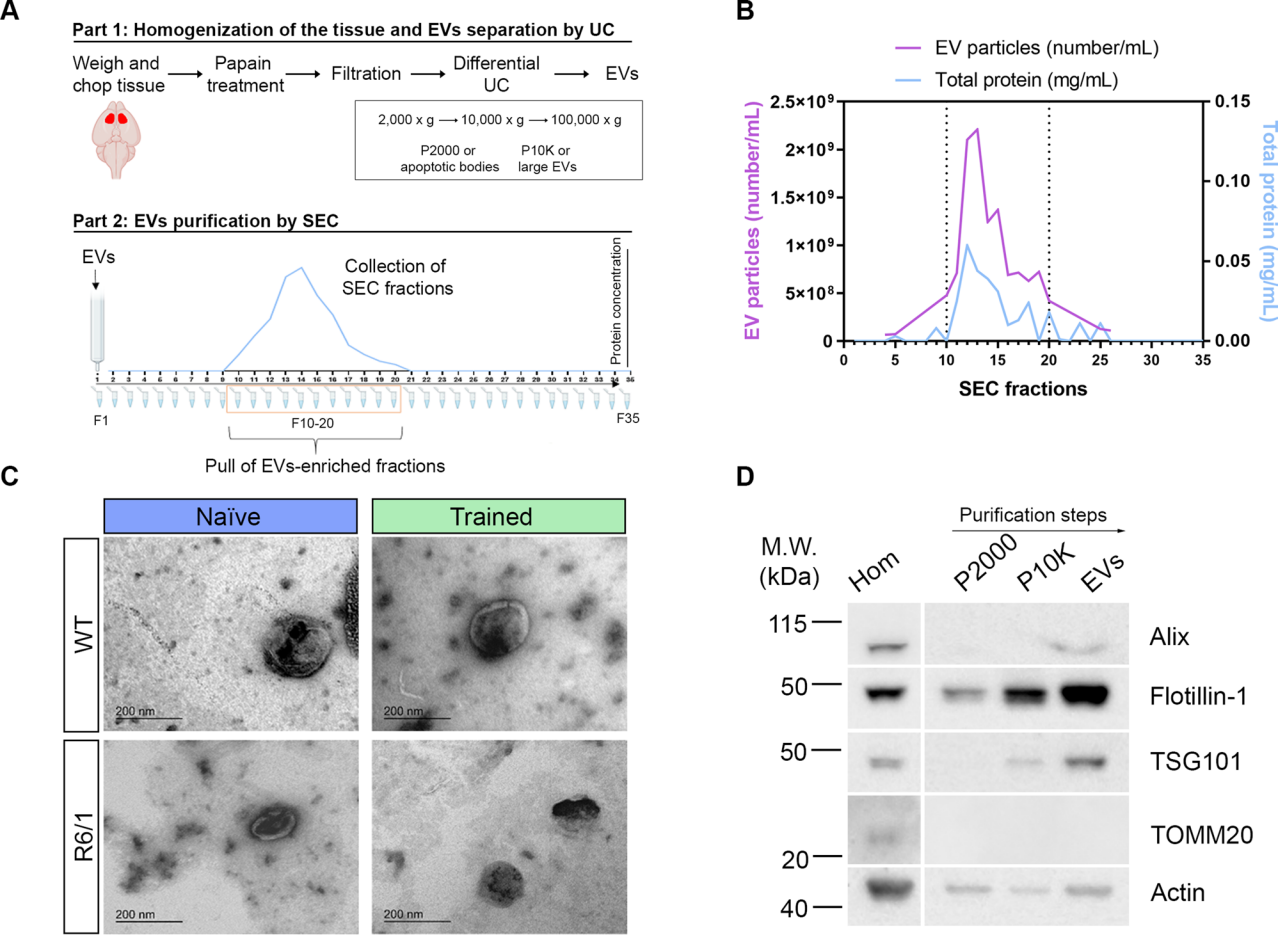
Isolation and purification of striatal EVs. **(A)** Schematic overview of EVs isolation from the striata. Striata was chopped and chemically digested, then homogenized and, cells, apoptotic bodies and large EVs were discarded by centrifugation. EVs were isolated from the supernatant by differential ultracentrifugation. EVs were then purified by SEC, and fractions 10 to 20 (peak in protein and particle concentration) were pulled together and considered as EV-enriched. **(B)** SEC elution profile. Total protein (blue) and EVs particle concentration (purple) was measured in each fraction by NanoDrop™ Spectrophotometer and NanoSight NS300, respectively. The peak of protein corresponds to the peak of EVs particles. **(C)** TEM micrographs of the vesicles show particles with the characteristic morphology and size of EVs, in the four groups (WT / R6/1 ± training). Images were visualized using negative staining. **(D)** Homogenates, apoptotic bodies (P2000), large microvesicles (P10K) and EVs were subjected to WB analysis with antibodies against EVs markers (Alix, Flotillin-1 and TSG101). TOMM20 is used as a negative EV control. Actin is used as a loading control for homogenates

Ninety minutes after the last rotarod trial, mice were sacrificed, and striatal tissue was dissected out. Then, EVs were isolated from the striatum of both WT and R6/1 mice, either naïve or trained, by a first step of sequential ultracentrifugation followed by a purification by SEC, obtaining a final pool of the fractions that correspond to the peak of protein (F10-20) (Fig. 2A). We showed that the protein peak overlapped the EVs peak, as judged by NTA analysis of the particle’s concentration combined with the protein measurements (Fig. 2B). Moreover, we confirmed the size and shape of small EVs using TEM, in our four conditions (WT / R6/1 ± training) (Fig. 2C). Furthermore, we characterized the different fractions obtained in the purification steps biochemically, by WB (homogenates, apoptotic bodies (P2000), large EVs (P10K) and small EVs). We confirmed that the EVs fraction was enriched in Alix, Flotillin-1 and TSG101, specific EVs markers, in comparison to the other fractions. Note that Alix and TSG101 are specific markers for exosomes, while Flotillin-1 can be found both in exosomes and in microvesicles [46]. The EVs fraction was also negative for the mitochondrial protein TOMM20 (Fig. 2D). Importantly, EVs fraction would contain EVs derived from all the neural cells naturally present in the striatum, cortical afferents, and striatal neurons but also astrocytes, oligodendrocytes, and microglia [47].

### Motor learning differently modulates the size and the concentration of striatal R6/1 EVs in comparison to WTs

**Fig. 3:**
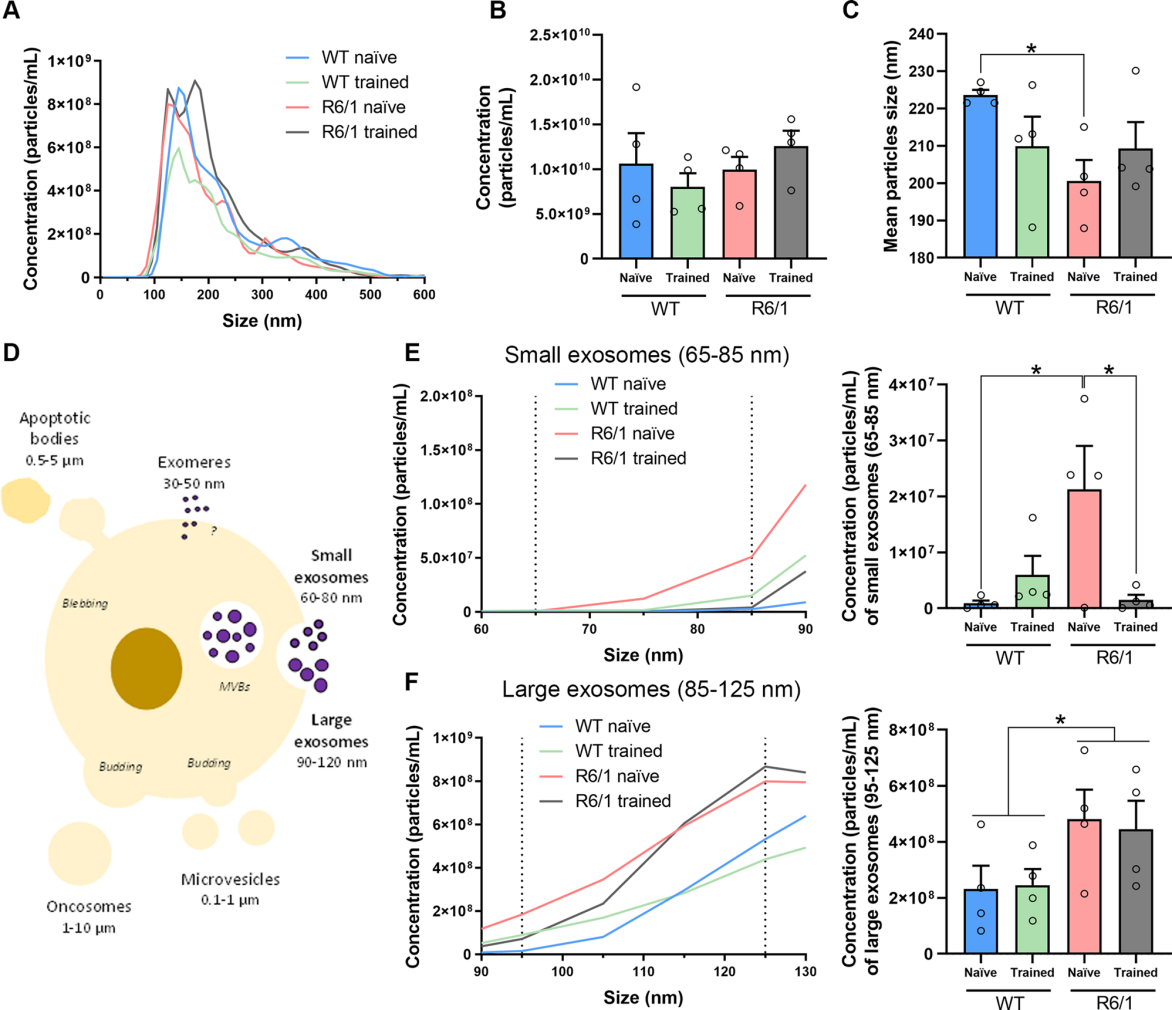
Striatal EVs from WT and R6/1 mice are differentially distributed in size and concentration. **(A)** Representative average curve of size distribution and particle concentration of the four different groups (WT / R6/1 ± training), by NTA analysis. Data is represented as the mean of the 4 animals per group and normalized by the tissue weight used for EVs-isolation. **(B)** Quantification of the total EVs particle concentration. **(C)** Quantification of the mean diameter (nm) of EVs particles. **(D)** Schematic representation of the different types of EVs, classified by size and biogenesis. **(E)** Vesicles ranging from 65 to 85 nm were selected (small exosomes) and concentration was represented. **(F)** Vesicles ranging from 95 to 125 nm were selected (large exosomes) and concentration was represented. Values are represented as mean ± SEM (*n* = 4). Data were analyzed by two-way ANOVA followed by Bonferroni’s post hoc test. (*P < 0.05)

To further characterize EVs populations in WT and R6/1 mice, with or without physical training, we assessed the distribution in size and particle concentration of the four groups by NTA (Fig. 3A). Although total particle concentration did not show differences between groups (Fig. 3B), we observed that R6/1 mice presented a lower mean size of the EVs particles than WT, and motor training mildly favored this size alteration in R6/1 (Fig. 3C). In the literature, many different types of EVs have been described, mostly classified by biogenesis and size as oncosomes, apoptotic bodies, microvesicles, large exosomes, small microvesicles and exomeres (Fig. 3D) [26]. Exclusively considering the size classification, our EVs samples mostly contain microvesicles (0.1–1 μm), large exosomes (90–120 nm), and small exosomes (60–80 nm), as reported by the size distribution of the four groups of EVs (Fig. 3A). Considering the particles in the range of 65 to 85 nm as small exosomes, we observed that R6/1 mice showed an increase in the concentration of this population in the striatum, in comparison to WT mice. This alteration was completely corrected when R6/1 mice learned the motor task (Fig. 3E). On the other hand, the concentration of the large exosome’s population (vesicles in the range of 85 nm to 125 nm) was higher in the R6/1 mice versus WT but was insensitive to motor skill learning in both genotypes (Fig. 3F).

### Striatal EVs proteomic signature reflects the signaling and metabolic alterations in R6/1 mice

**Fig. 4:**
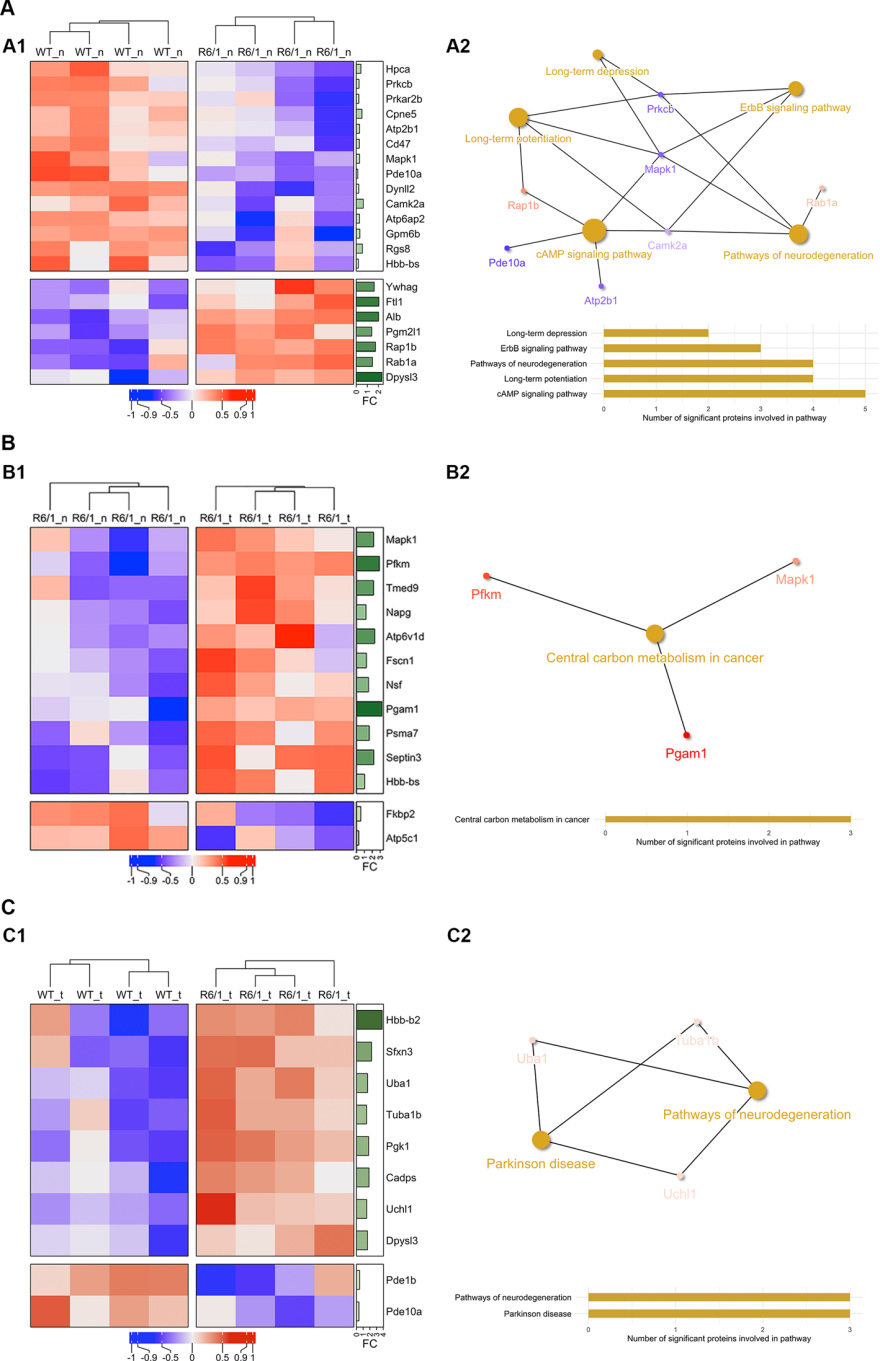
Striatal EVs from naïve or trained WT and R6/1 mice present a differential proteomic signature that results in biological pathways’ alterations. (**A**) Pairwise comparison of naïve WT and R6/1 mice striatal EVs. (A1) Heatmap showing the differentially expressed proteins in WT and R6/1 mice derived striatal EVs (*n* = 4 per group). (A2) Network plot show in yellow the significant pathways that are altered considering the proteomic content of EVs. (**B**) Pairwise comparison of naïve R6/1 and trained R6/1 striatal EVs. (B1) Heatmap showing the differentially expressed proteins in naïve R6/1 and trained R6/1 mice striatal EVs (*n* = 4 per group). (B2) Network plot show in yellow the significant pathways that are altered considering the proteomic content of EVs. (**C**) Pairwise comparison of trained WT and R6/1 striatal EVs. (C1) Heatmap showing the differentially expressed proteins in WT trained and R6/1 trained mice striatum-EVs (*n* = 4 per group). (C2) Network plot show in yellow the significant pathways that are altered considering the proteomic content of EVs. In all cases, statistically significant overexpressed proteins are depicted in red, whereas proteins that are underrepresented are shown in blue. In the right annotation the fold change (FC) is displayed in green as a bar plot for each of the proteins (the darker the color, the higher the FC value). FC is calculated as 2^(mean1-mean2). Proteins were considered significant when the p value was under 0.05 in a t-test and a FC of less than 0.33 or above 1.7

To investigate whether WT and R6/1 mice striatal EVs differ in their protein cargo, we assessed the proteome of naïve WT and R6/1 striatal EVs. When we compared the whole proteomic signature, we found a significant separation of the two groups in the PCA, constructed with top variables based on a PLS-DA analysis (Figure S1A). Indeed, the heatmap summarizes all the differentially expressed proteins in striatal EVs from the two naïve groups (Fig. 4A_1_). Remarkably, the most overexpressed proteins in R6/1 striatal EVs were ferritin, dihydropyrimidinase-like 3 protein (DPYSL3) and albumin.

Using the KEGG database [48–50] with all the protein data, we extracted the biological pathways that were significant: long-term potentiation, long-term depression, ErbB/ERK signaling pathway, cAMP signaling pathway and pathways of neurodegeneration (Fig. 4A_2_, Supplementary Table 1). Interestingly, the alteration of these pathways has a crucial role in the pathogenesis of HD [51, 52].

To study the effect of motor learning on R6/1 mice, we compared the protein cargo of striatal EVs from naïve or trained R6/1 mice. Again, PCA plots revealed that motor training was sufficient to modulate the protein content of EVs in R6/1 mice (Figure S1B). The heatmap showed a general upregulation of differentially expressed proteins after the rotarod training in the R6/1 animals (Fig. 4B_1_). In this case, we found significant alterations in metabolic pathways (Fig. 4B_2_, Supplementary Table 2). Indeed, the proteins that presented higher levels in striatal R6/1 EVs were the muscle isoenzyme phosphofructokinase (PFKM) and phosphoglycerate mutase 1 (PGAM1), both involved in the glycolytic pathway. Interestingly, decreased levels of PGAM1 have been found in the brain of HD patients (Huntington’s Disease_CNS-Brain (MMHCC)_GSE857, Harmonizome 3.0), revealing a potential beneficial function of motor learning in the modulating the molecular composition of striatal EVs.

Hence, we showed that motor skill learning did not mask HD alterations in metabolism [53] in the EVs from the trained R6/1 mice.

To investigate whether motor learning could also influence striatal EVs protein cargo in WT mice, we assessed EVs protein content of naïve and trained WT striatal EVs. PCA plot revealed that motor learning could not separate striatal EVs from naïve or trained WT mice, as judged by the lack of sample group clustering (Figure S1C). However, pairwise comparisons of the proteomic data of naïve and trained WT striatal EVs identified several differentially expressed proteins in EVs after the training (Figure S2). Although we did not find significant alterations in general biological pathways (Supplementary Table 3), we observed that after learning the motor task, there was a lower expression of proteins involved in protein translation, such as seryl-aminoacyl-tRNA synthetase (SerRS) [54], or in plasticity and metabolism such as synaptosomal-associated protein 25 (SNAP25), phosphoglycerate kinase 1 (PGK1), protein kinase cAMP dependent regulatory (PRKAR2B) and nipsnap2 homolog 2 (NIPSNAP2) [55–58] (Figure S2).

Interestingly, when we compared trained WT and R6/1 groups, PCA plot confirmed that the two groups did not differ in the protein content (Figure S1D). The heatmap revealed mostly upregulated proteins (Fig. 4C_1_), that resulted in an alteration in pathways related with neurodegeneration and Parkinson’s disease (Fig. 4C_2_, Supplementary Table 4).

**Fig. 5:**
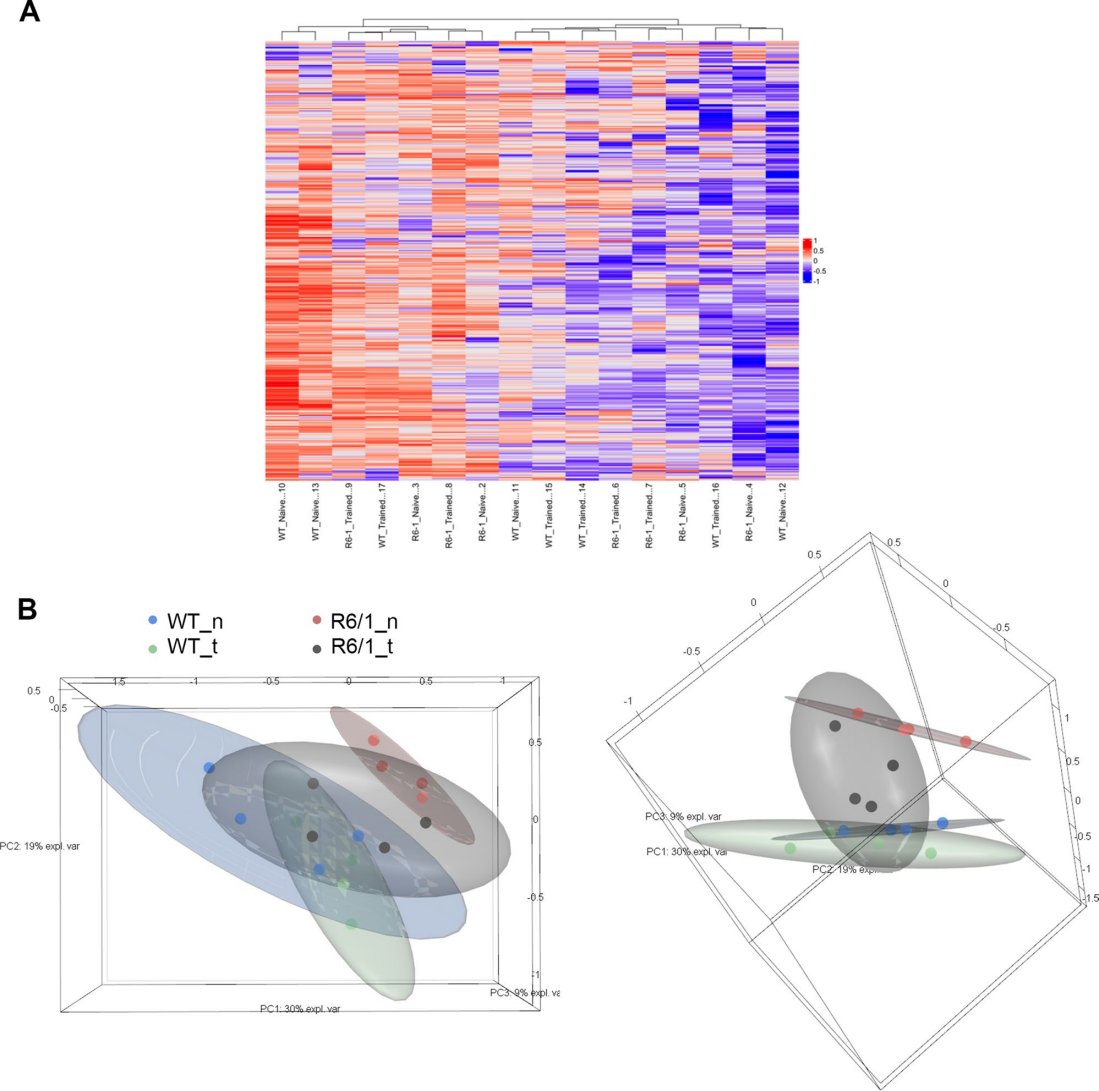
R6/1 mice striatal EVs get more similar to WT after motor learning. **(A)** Heatmap showing all the proteins detected in striatum EVs in the four animals per condition, with the method used (LC-MS/MS). Overexpressed proteins are depicted in red, whereas proteins that are underrepresented are shown in blue. **(B)** PCA-3D model plot constructed with top variables based on a PLS-DA analysis shows clear clustering of naïve WT (WT_n) and naïve R6/1 (R6/1_n) mice striatal EVs, regarding EVs protein composition, but no separation between the other groups. To construct the model, the whole list of proteins –whether significantly altered or not between groups– was used. Component 1 stand for an 30% of variance, component 2 for a 19% and component 3 for a 9%. In addition, surrounding ellipses represent the 95% confidence interval for each group

When we plotted the four groups together (WT / R6/1 ± training), the PCA in three dimensions (3D) completely clustered EVs content per genotype (naïve WT and naïve R6/1) but not by motor learning, meaning that acquiring the task brings closer the protein content of R6/1 EVs to either the naïve or the trained WT EVs (Fig. 5).

Indeed, the pairwise comparison of naïve WT and trained R6/1 derived striatal EVs showed no clustering regarding EVs protein content, suggesting, again, an evident effect of motor training in R6/1 mice EVs proteomic composition (Figure S3).

### Motor learning training restores normal levels of ERK2 and β-globin proteins in striatal EVs and has a mild effect on cell survival and synaptic plasticity pathways

**Fig. 6:**
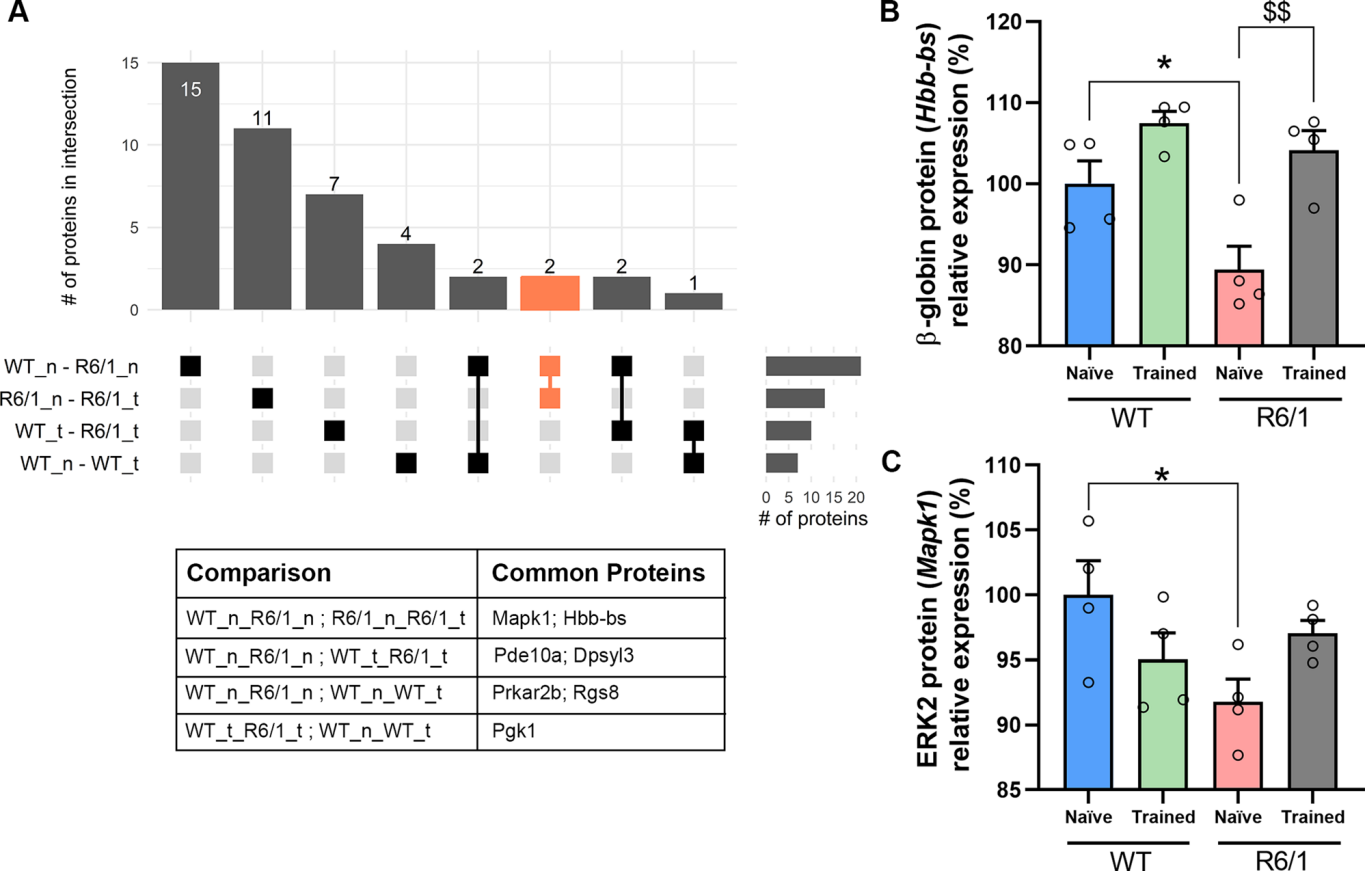
Motor learning restores normal levels of ERK2 and β-globin in R6/1 mice striatal EVs. **(A)** UpSet plot shows the number of proteins in striatal EVs that overlap among the four comparisons: naïve WT vs. naïve R6/1 (WT_n_R6/1_n), naïve R6/1 vs. trained R6/1 (R6/1_n_R6/1_t); trained WT vs. trained R6/1 (WT_t_R6/1_t), and naïve WT vs. trained WT (WT_n_WT_t). The comparison of interest is shown in orange. The table indicates which proteins are overlapping in each case. **(B)** Quantification of ERK2 levels in striatal EVs. **(C)** Quantification of beta-globin levels in striatal EVs. Values are represented as mean ± SEM (*n* = 4). Data were analyzed by two-way ANOVA followed by Bonferroni’s post hoc test. (*P < 0.05 vs. WT naïve; ^$$^*P <* 0.01 vs. R6/1 naïve)

To further investigate the potential beneficial role of motor learning via EVs, we assessed the levels of the proteins that were shared between the four groups of study. Using an UpSet plot, we reported two proteins that were shared in both comparisons of interest, that resulted to be ERK2 (*Mapk1*) and β-globin (*Hbb-bs*) (Fig. 6A). We observed that both proteins were reduced in striatal EVs from naïve R6/1 mice, but motor learning reverted their levels (Fig. 6B & C). These results highly indicate that learning a motor task affects directly the striatal EVs content and modulate specific ERK2 (Mapk1) and β-globin (Hbb-bs) signaling deficits in an HD mouse model.

**Fig. 7:**
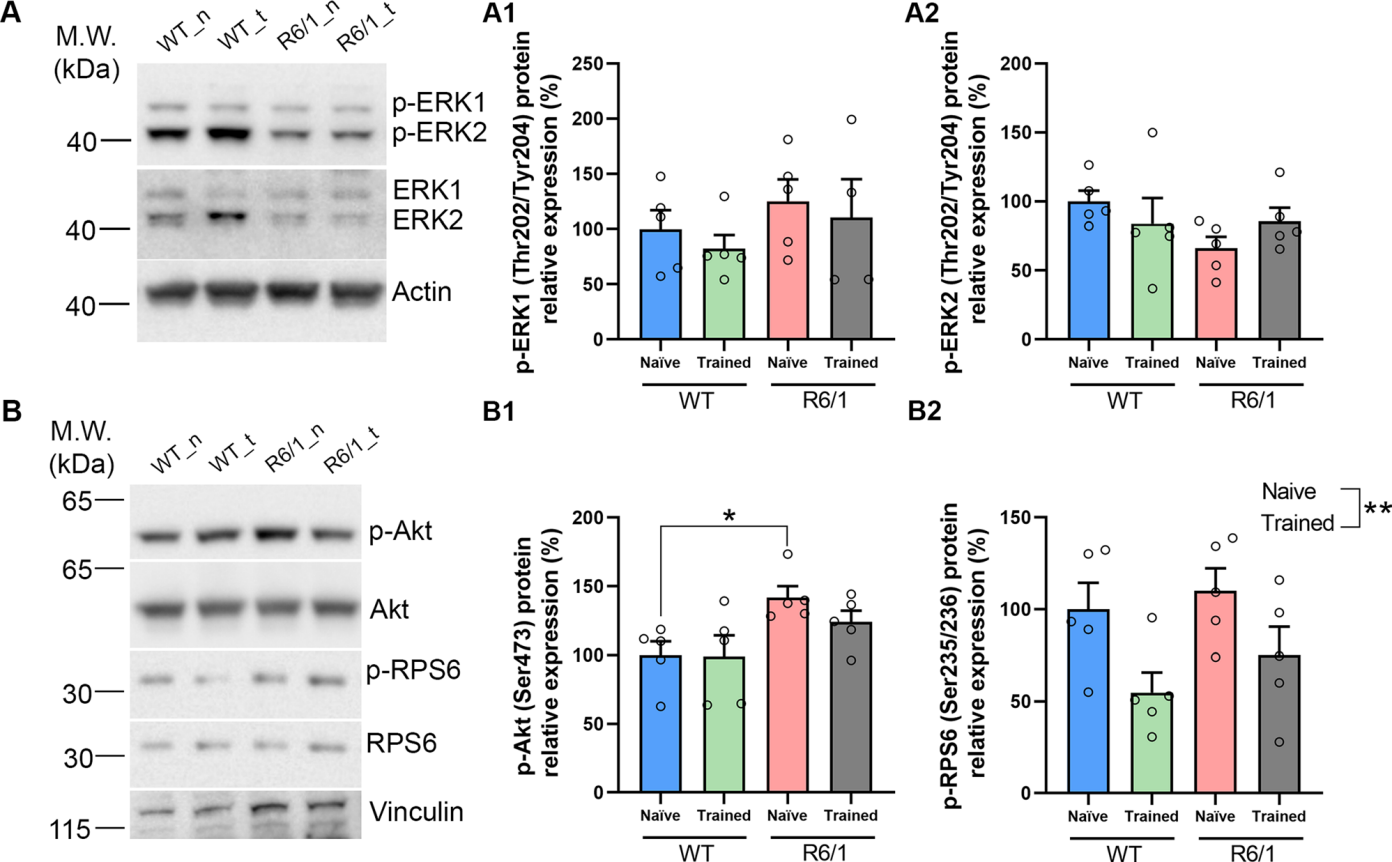
Motor learning mildly restores the physiological Akt phosphorylation in R6/1 mice. Striatal homogenates from naïve or trained WT and R6/1 mice, were subjected to WB analysis. Actin or vinculin are used as loading controls. **(A)** Representative immunoblots show phospho-ERK1/2 Thr202/Tyr204 and total ERK. Densitometric analysis of (A1) phospho-ERK1 and (A2) phosphor-ERK2. **(B)** Representative immunoblots show phospho-Akt Ser473, phospho-RPS6 Ser235/236, total Akt and total RPS6. Densitometric analysis of (B1) phospho-Akt and (B2) phospho-RPS6. Data were analyzed by two-way ANOVA followed by Bonferroni’s post hoc test (*P < 0.05 vs. WT naïve)

Since R6/1 mice striatal EVs showed a disruption in biological pathways involved in synaptic plasticity and cell survival [51, 52] (Fig. 4A_2_), we investigated whether we could observe these effects in the recipient structure, the striatum, from the same animals, by WB. We could not observe significant differences in survival/plasticity readouts [59–61], such as the phosphorylated levels of ERK (Fig. 7A) in the striatal homogenates of the four groups (WT / R6/1 ± training). Although the levels of phospho-ERK1 remained unaltered between conditions (Fig. 7A_1_), we observed non-significant mild tendencies in the recovery of phospho-ERK2 after training in the R6/1 mouse group (Fig. 7A_2_), in line with our observations of the ERK2 levels in striatal EVs (Fig. 6C). Interestingly, we confirmed the expected elevated levels of phospho(S473)-Akt in R6/1 mice striatal lysates [29, 62], and this was partially corrected in the R6/1 mice after learning a motor skill (Fig. 7B_1_). Finally, we observed that phosphorylation of RPS6 (Ser235/236) was sensitive to motor learning in both WT and R6/1 mice, independently of their genotype (Fig. 7B_2_).

These results indicate that motor learning tasks in R6/1 mice directly influences the striatal EVs composition, which could affect their function, and therefore might have a resilient impact on cell survival and synaptic plasticity pathways.

## Discussion

This study describes for the first time that the R6/1 mouse model presents a specific striatal EVs profile with a proteomic content that reflects both the signaling and the synaptic alterations described in HD. Moreover, exposing R6/1 mice to a motor learning task that activates the cortico-striatal pathway using rotarod, significantly changed the striatal EVs signature and reversed some of the protein deficiencies, highly indicating a resilience-inducing role of motor training in HD via transcellular communication.

Here, we reported that R6/1 mice showed a different striatal EVs profile, in terms of size and concentration. R6/1 striatal EVs presented higher concentrations of both small and large exosomes. Although Ananbeh et al. (2022) did not find significant differences in the size of EVs isolated from blood plasma of pig models of HD [63], this difference could be explained by the EVs source, as brain-derived EVs might represent only a minority of all plasma vesicles [64]. Indeed, this could indicate that this size alteration is more specific to neural-EVs derived from the striatum. Furthermore, this higher exosome concentration in R6/1 mice striatum, could be due to an increase in exosome secretion. Indeed, in neurodegenerative diseases, including HD, the impairment in the endo-lysosomal pathway results in an increased secretion of exosomes [65, 66]. Interestingly, rotarod training of the R6/1 mice, nearly palliated EVs-size alterations, highly indicating an adaptability of the EVs signature to a physical training that involves motor learning. Indeed, motor learning processes activate the cortico-striatal synaptic pathway, and it has been described that neural EVs are released in response to synaptic glutamatergic activity [67, 68] and have a role modulating synaptic plasticity [24].

Our proteomic results also showed that R6/1 mice had alterations in the protein content of striatal EVs, compared to WT. The most upregulated proteins in R6/1 striatal EVs were ferritin, DPYSL3 and albumin. In HD patients, there are high levels of ferritin [69], and this has been associated with cell death by ferroptosis [70]. DPYSL3 has been linked to elevated mhtt levels in human fibroblasts samples [71]. Moreover, the presence of increased levels of albumin in striatal EVs could be a sign of leakage due to a blood-brain barrier permeability perturbations [72] or to a microglial activation [73]. Interestingly, striatal R6/1 EVs-proteome evoked the alterations in synaptic and signaling pathways that have been described in HD, such as long-term potentiation and depression [74], cAMP signaling pathway [75], ErBB/ERK signaling pathways [52] and even pathways of neurodegeneration. These results reinforce the idea that EVs are active contributors to the pathogenesis of the disease [76], as they are sufficient to modulate signaling pathways in the neighboring cells due to their content.

Intriguingly, neither htt nor mhtt protein was detected in striatal EVs by LC-MS/MS. This could be explained by the result of the trypsinization of EVs, that is very well influenced by the aggregating nature of this protein [29, 77]. Ananbeh et al. (2022) [63] found htt protein in small EVs from plasma of transgenic knock-in pig models of HD, and from human HD patients. In line with this, Miguez et al. (2023) [22] isolated EVs derived from HD-derived human NPCs lines and used them to treat mouse primary striatal neurons. After 24 h, they observed the presence of soluble mhtt in mouse striatal neurons by immunocytochemistry and TEM immunogold. However, in both studies [22, 63], whether mhtt is loaded in EVs or co-isolated with EVs remains unknown.

Furthermore, motor learning changed the proteomic profile of striatal EVs significantly in the R6/1 mice. We included the accelerating rotarod test because it involves physical activity and a motor learning curve in contrast to the voluntary running wheel, for example, where the animals run freely with no control in the period of the physical activity. In line with the described alterations in oxidative phosphorylation [78], oxidative stress [79] and mitochondrial functioning [80] in HD, we found that in EVs there were alterations in proteins involved in metabolism and in the central carbon metabolism in cancer. In physiological conditions, the major pathway to get ATP is oxidative phosphorylation. This process is very slow, so in pathological conditions, such as in neurodegeneration, cells use a faster way to produce ATP by glycolysis [81, 82]. This Warburg-like metabolic transformation has been recently reported in other neurodegenerative disorders, such as Alzheimer’s disease (AD), and underlies neuronal degeneration [83]. Therefore, this observation of alterations in the central carbon metabolism is in accordance with the compensatory shift in brain energy metabolism that happens in the striatum of HD patients [84, 85].

Overall, we reported upregulated levels of metabolic proteins after motor learning in striatal EVs of R6/1 mice, which could indicate that R6/1 mice have higher energetic requirements than WT during physical activity. In contrast, in WT striatal EVs physical training induced a downregulation of proteins involved in metabolism, such as SNAP25 [55], PGK1 [56], PRKAR2B [57] and NIPSNAP2 [58]. This contrary effect of training in WT and R6/1 mice seem to evoke an impaired homeostatic response to training in the R6/1 mice.

Strikingly, motor learning seemed to regulate crucial pathways of neurodegeneration in the protein content of striatal EVs. Considering the proteome profile of striatal EVs, we found that WT and R6/1 derived EVs profiles were completely different, but EVs from R6/1 mice subjected to rotarod training got closer to WT EVs. Again, this reinforces the idea that motor learning could interfere effectively the EVs signaling in the striatum. The differential content and size of the EVs in this HD mouse model at 2 months could be compared with older animals’ samples to establish a disease progression profile, to finally correlate them with eventual peripheral biomarkers in HD patients. However, it is unknown whether this accelerating rotarod effect seen at 2 months old animals could be reproduced in older ones, mostly because their more severe motor deficits could impair the test performance.

In addition, we reported reduced levels of β-globin in R6/1 mice striatal EVs. HD pathophysiology includes iron dysregulation, which can promote iron-deficiency anemia [86]. Neuronal hemoglobin has a crucial role in the maintenance of normal mitochondrial functioning in the brain [87]. We showed that β-globin levels in striatal EVs were completely compensated in R6/1 mice subjected to a motor skill learning. This is in line with Dehghan et al. (2021), that reported increased levels of β-globin in mice brain after physical training [88].

Furthermore, R6/1 striatal EVs showed reduced levels of ERK2 versus WT EVs. Downregulated levels of ERK2 have been reported in the striatum of HD human post-mortem brains and in mouse models of the disease, and is linked to a synaptic dysfunction [89, 90]. We observed that this deficiency is transferred via EVs in the striatum, and strikingly, physiological levels of ERK2 were partially restored in R6/1 mice subjected to a motor learning, similar to the synaptic effect of neural EVs [24, 91]. This is in line with Taylor et al. (2012), who showed that training upregulated ERK1/2 signaling in skeletal muscle because of hypertrophic adaptations [92]. More specifically in neural cells, physical exercise has been shown to promote the functional recovery of neurons after stroke and inhibits apoptosis in diabetes via ERK [93, 94]. As ERK activation has been proposed to be protective in HD [52], we suggest that the modulation of its levels in EVs after acute or even long-term training might induce resilience for the HD pathology. Moreover, since neural EVs mediate synaptic plasticity [24], this could even ameliorate the HD-related synaptic deficiencies. Setting EVs apart, in striatal cells of HD models it has been described that ERK2 phosphorylation is decreased [90, 95]. Interestingly, we observed a non-significant tendency to compensate phospho-ERK2 decrease after R6/1 physical training. This effect seemed to be specific of ERK2, as no tendencies were observed in ERK1 phosphorylation.

Furthermore, we reported reduced levels of phospho-RPS6 in the striatum after motor still learning involving acute training, as previously described by others in the liver [96] and in the muscle [97], but no differences were found in R6/1 mice. In the case of Akt, we confirmed that R6/1 mice presented an hyperphosphorylation in the striatum, as observed by Saavedra et al. (2010) [62] and Martín-Flores et al. (2020) [29]. This activation has been suggested to be a short-term pro-survival response against mhtt toxicity [98] which could be detrimental long-term for cell survival and synaptic function [99]. Strikingly, this overactivation of Akt was partially reduced in R6/1 mice exposed to motor learning, suggesting again that acquiring a motor skill could contribute to a resilience response and could modulate the pathogenesis of HD.

Overall, our results indicate that striatal R6/1 EVs show alterations in size and in the proteomic signature, which outline the signaling and metabolic alterations present in HD, opening subsequent studies to further characterize EVs specifically on tissue to acquire a better understanding of neurodegeneration. Moreover, our results put motor learning processes as modulators of striatal EVs profile, which could in turn in harmonize cell to cell communication in the striatum, with the ultimate goal of a disease modifying therapeutic approach.

### Electronic supplementary material

Below is the link to the electronic supplementary material.

Supplementary Material 1

## Data Availability

Mass-spectrometry data and search results files were deposited in the Proteome Xchange Consortium via the JPOST partner repository (https://repository.jpostdb.org) (Okuda S, Watanabe Y, Moriya Y, Kawano S, Yamamoto T, Matsumoto M, Takami T, Kobayashi D, Araki N, Yoshizawa AC et al.: jPOSTrepo: an international standard data repository for proteomes. Nucleic Acids Res 2017, 45(D1):D1107-D1111) with the identifier PXD041680 for ProteomeXchange and JPST002132 for jPOST (for reviewers: https://repository.jpostdb.org/preview/170310131463ea007f81c18; Access key: 3507).

## Abbreviations

AD: Alzheimer’s disease
CAG: Cytosine-Adenine-Guanine
DPYSL3: Dihydropyrimidinase-like 3 protein
EVs: Extracellular vesicles
GSEA: Gene site enrichment analysis
HD: Huntington’s disease
Htt: Huntingtin
Mhtt: Mutant huntingtin
MS: Mass spectrometry
NIPSNAP2: Nipsnap2 homolog 2
NTA: Nanoparticle tracking analysis
PBS: Phosphate-buffered saline
PCA: Principal component analysis
PFKM: Muscle isoenzyme phosphofructokinase
PGAM1: Phosphoglycerate mutase 1
PGK1: Phosphoglycerate kinase 1
PLS-DA: Partial least square discriminant analysis
PolyQ: Poly-glutamine
PRKAR2B: Protein kinase cAMP dependent regulatory
SEC: Size exclusion chromatography
SEM: Standard error of the mean
SeRS: Seryl-aminoacyl-tRNA synthetase
SNAP25: Synaptosomal-associated protein 25
TEM: Transmission electron microscopy
VIP: Importance projection metric
WB: Western Blot
WT: Wild type
