# Supplementary material for: Motor skill learning modulates striatal extracellular vesicles’ content in a mouse model of Huntington’s disease
Source: Cell Commun Signal. 2024 Jun 11;22:321. doi: 10.1186/s12964-024-01693-9 (PMC11167907; doi:10.1186/s12964-024-01693-9)
Supplement: Supplementary file 1 — Supplementary Material 1 [file 12964_2024_1693_MOESM1_ESM.pdf]

SUPPLEMENTARY FIGURES AND TABLES:

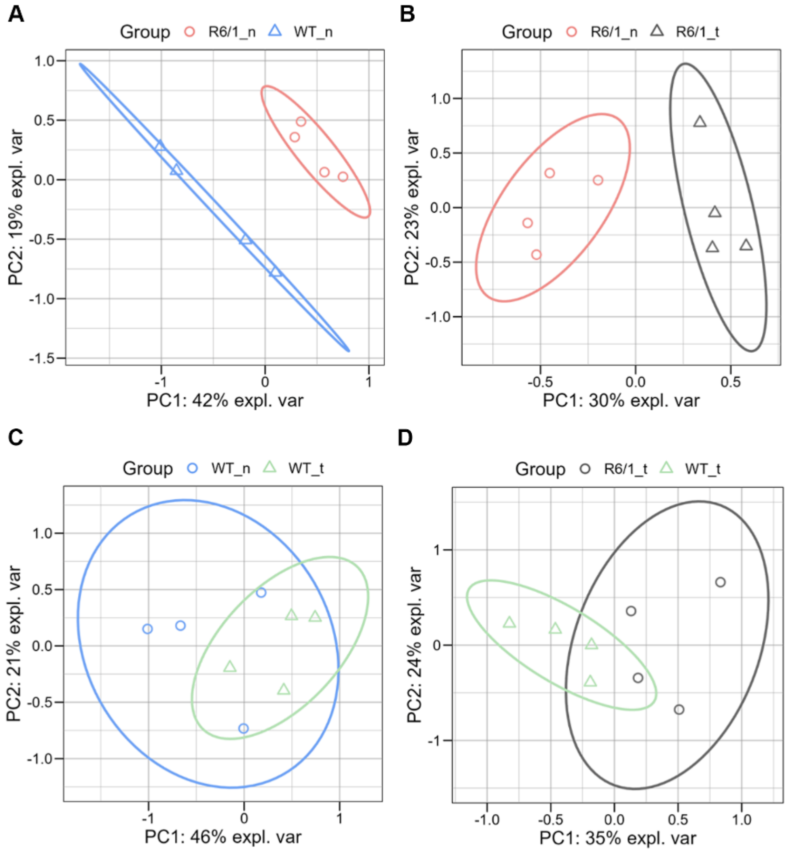

Supplementary Figure 1. Motor learning alters striatal EVs composition in R6/1 mice. PCA plot constructed with top variables on the basis of a PLS-DA analysis, based on striatal EVs protein composition of **(A)** naïve WT (in blue, WT_n) and trained WT (in green, WT_t), **(B)** naïve WT (in blue) and naïve R6/1 (in red, R6/1_n), **(C)** naïve R6/1 (in red) and trained R6/1 (in gray, R6/1_t) and **(D)** trained WT (in green) and trained R6/1 (in gray). To construct the model, the whole list of proteins –whether significantly altered or not between groups– was used. Component 1 (46% variance in A, 42% in B, 30% in C and 35% in D) and component 2 (21% variance in A, 19% in B, 23% in C and 24% in D) explain intra-group variability and separate or not the samples belonging to each group. In addition, surrounding ellipses represent the 95% confidence interval for each group.

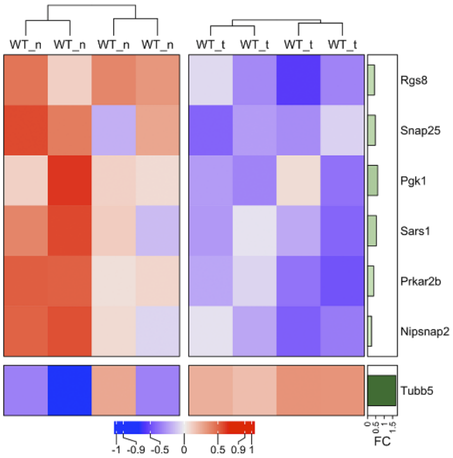

Supplementary Figure 2. Motor learning modulates the proteomic signature of striatal EVs in WT mice. Pairwise comparison of naïve or trained WT mice striatal EVs. Heatmap showing the differentially expressed proteins in naïve or trained WT mice derived striatal EVs. Significantly overexpressed proteins in the trained WT group in comparison to the naïve WT group are depicted in red, whereas proteins that are underrepresented in the trained WT are shown in blue. In the right annotation the log2 fold change (FC) is displayed as a bar plot for each of the proteins.

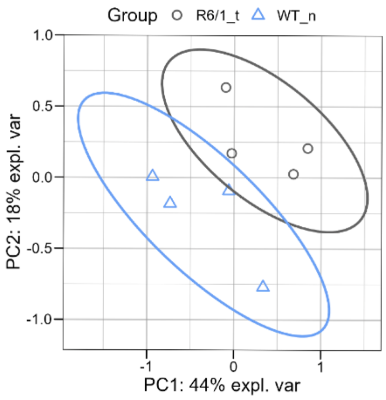

Supplementary Figure 3. PCA plot does not separate naïve WT and trained R6/1 striatal EVs by protein content. PCA plot based on striatal EV protein composition of naïve WT (in blue, WT_n) and trained R6/1 (in gray, R6/1_t). To construct the model, the whole list of proteins –whether significantly altered or not between groups– was used. Component 1 (44% variance) and component 2 (18% variance) explain intra-group

variability and separate or not the samples belonging to each group. In addition, surrounding ellipses represent the 95% confidence interval for each group.

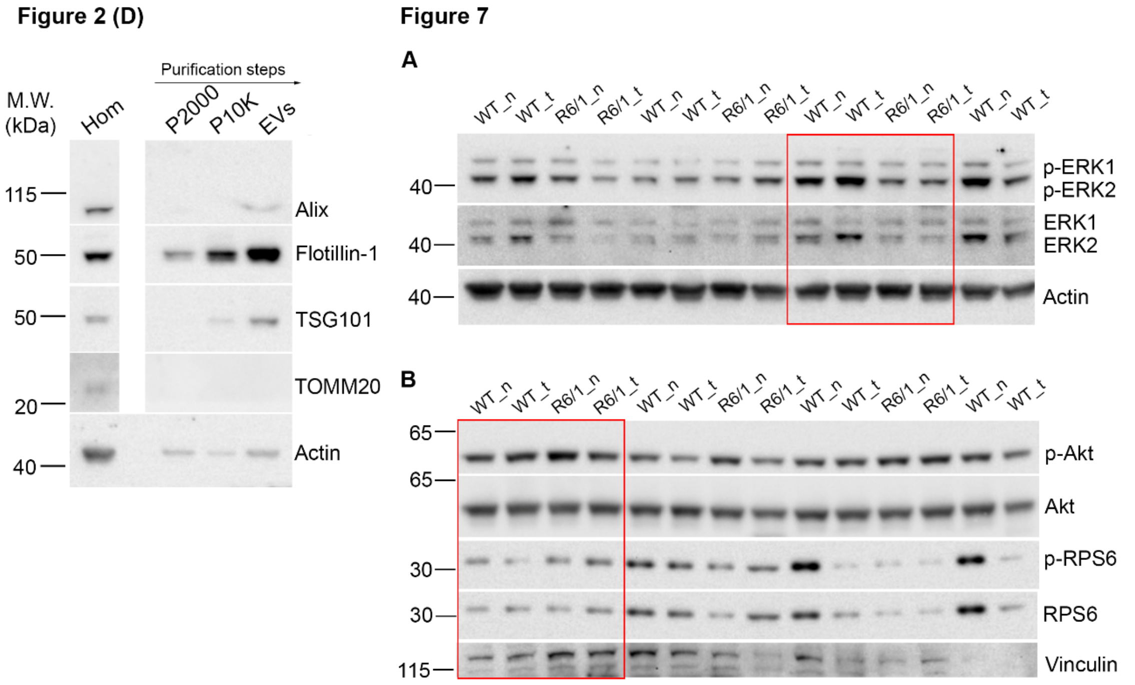

Supplementary Figure 4. Full-length blots for Figures 2 and 7. Red rectangles indicate the part of each blot that was used in the panels of the figures. Molecular weights are indicated.

Supplementary Table 1. Biological pathways analysis of naïve WT and R6/1 striatal EVs using the KEGG database of mice proteome. Frequency indicates the number of proteins significantly altered involved in a particular biological pathway.

**Biological pathways**	**Frequency**	**Protein ID**
cAMP signaling pathway	5	Atp2b1; Camk2a; Mapk1; Pde10a; Rap1b
Long-term potentiation	4	Camk2a; Mapk1; Prkcb; Rap1b
Pathways of neurodegeneration	4	Camk2a; Mapk1; Prkcb; Rab1a
Adrenergic signaling in cardiomyocytes	3	Atp2b1; Camk2a; Mapk1
Aldosterone synthesis and secretion	3	Atp2b1; Camk2a; Prkcb
Calcium signaling pathway	3	Atp2b1; Camk2a; Prkcb
Chemical carcinogenesis	3	Mapk1; Mapk1; Prkcb
Chemokine signaling pathway	3	Mapk1; Prkcb; Rap1b
Cholinergic synapse	3	Camk2a; Mapk1; Prkcb

Circadian entrainment	3	Camk2a; Mapk1; Prkcb
Cushing syndrome	3	Camk2a; Mapk1; Rap1b
ErbB signaling pathway	3	Camk2a; Mapk1; Prkcb
Focal adhesion	3	Mapk1; Prkcb; Rap1b
Glioma	3	Camk2a; Mapk1; Prkcb
GnRH signaling pathway	3	Camk2a; Mapk1; Prkcb
HIF-1 signaling pathway	3	Camk2a; Mapk1; Prkcb
Lipid and atherosclerosis	3	Camk2a; Mapk1; Rap1b
MAPK signaling pathway	3	Mapk1; Prkcb; Rap1b
Melanogenesis	3	Camk2a; Mapk1; Prkcb
Neurotrophin signaling pathway	3	Camk2a; Mapk1; Rap1b
Oocyte meiosis	3	Camk2a; Ywhag; Mapk1
Oxytocin signaling pathway	3	Camk2a; Mapk1; Prkcb
Pancreatic secretion	3	Atp2b1; Prkcb; Rap1b
Pathways in cancer	3	Camk2a; Mapk1; Prkcb
Proteoglycans in cancer	3	Camk2a; Mapk1; Prkcb
Rap1 signaling pathway	3	Mapk1; Prkcb; Rap1b
Ras signaling pathway	3	Mapk1; Prkcb; Rap1b
African trypanosomiasis	2	Prkcb; Hbb-bs
AGE-RAGE signaling pathway in diabetic complications	2	Mapk1; Prkcb
Aldosterone-regulated sodium reabsorption	2	Mapk1; Prkcb
Amphetamine addiction	2	Camk2a; Prkcb
Autophagy	2	Mapk1; Rab1a
Axon guidance	2	Camk2a; Mapk1
B cell receptor signaling pathway	2	Mapk1; Prkcb
cGMP-PKG signaling pathway	2	Atp2b1; Mapk1
Choline metabolism in cancer	2	Mapk1; Prkcb
Coronavirus disease	2	Mapk1; Prkcb
Diabetic cardiomyopathy	2	Camk2a; Prkcb
Dopaminergic synapse	2	Camk2a; Prkcb
EGFR tyrosine kinase inhibitor resistance	2	Mapk1; Prkcb
Endocrine and other factor-regulated calcium reabsorption	2	Atp2b1; Prkcb
Fc gamma R-mediated phagocytosis	2	Mapk1; Prkcb
Gap junction	2	Mapk1; Prkcb
Gastric acid secretion	2	Camk2a; Prkcb
Glutamatergic synapse	2	Mapk1; Prkcb
GnRH secretion	2	Mapk1; Prkcb

Growth hormone synthesis, secretion, and action	2	Mapk1; Prkcb
Hepatitis B	2	Mapk1; Prkcb
Hepatitis C	2	Ywhag; Mapk1
Hepatocellular carcinoma	2	Mapk1; Prkcb
Human cytomegalovirus infection	2	Mapk1; Prkcb
Human immunodeficiency virus 1 infection	2	Mapk1; Prkcb
Inflammatory mediator regulation of TRP channels	2	Camk2a; Prkcb
Influenza A	2	Mapk1; Prkcb
Insulin secretion	2	Camk2a; Prkcb
Insulin signaling pathway	2	Prkar2b; Mapk1
Leishmaniasis	2	Mapk1; Prkcb
Leukocyte trans endothelial migration	2	Prkcb; Rap1b
Long-term depression	2	Mapk1; Prkcb
Metabolic pathways	2	Pde10a; Pgm2l1
MicroRNAs in cancer	2	Mapk1; Prkcb
Mineral absorption	2	Atp2b1; Ftl1
Morphine addiction	2	Prkcb; Pde10a
mTOR signaling pathway	2	Mapk1; Prkcb
Natural killer cell mediated cytotoxicity	2	Mapk1; Prkcb
Necroptosis	2	Camk2a; Ftl1
Neutrophil extracellular trap formation	2	Mapk1; Prkcb
Non-small cell lung cancer	2	Mapk1; Prkcb
Parathyroid hormone synthesis, secretion, and action	2	Mapk1; Prkcb
PI3K-Akt signaling pathway	2	Ywhag; Mapk1
Platelet activation	2	Mapk1; Rap1b
Renal cell carcinoma	2	Mapk1; Rap1b
Retrograde endocannabinoid signaling	2	Mapk1; Prkcb
Salivary secretion	2	Atp2b1; Prkcb
Salmonella infection	2	Mapk1; Dynll2
Serotonergic synapse	2	Mapk1; Prkcb
Sphingolipid signaling pathway	2	Mapk1; Prkcb
Thyroid hormone signaling pathway	2	Mapk1; Prkcb
Thyroid hormone synthesis	2	Alb; Prkcb
Tuberculosis	2	Camk2a; Mapk1

Vascular smooth muscle contraction	2	Mapk1; Prkcb
VEGF signaling pathway	2	Mapk1; Prkcb
Viral carcinogenesis	2	Ywhag; Mapk1
Wnt signaling pathway	2	Camk2a; Prkcb
Acute myeloid leukemia	1	Mapk1
Adherens junction	1	Mapk1
Alcoholism	1	Mapk1
Alzheimer disease	1	Mapk1
Amoebiasis	1	Prkcb
Amyotrophic lateral sclerosis	1	Rab1a
Apelin signaling pathway	1	Mapk1
Apoptosis	1	Mapk1
Bladder cancer	1	Mapk1
Breast cancer	1	Mapk1
C-type lectin receptor signaling pathway	1	Mapk1
Carbohydrate digestion and absorption	1	Prkcb
Cell cycle	1	Ywhag
Cellular senescence	1	Mapk1
Central carbon metabolism in cancer	1	Mapk1
Chagas disease	1	Mapk1
Chronic myeloid leukemia	1	Mapk1
Colorectal cancer	1	Mapk1
ECM-receptor interaction	1	Cd47
Endocrine resistance	1	Mapk1
Endometrial cancer	1	Mapk1
Estrogen signaling pathway	1	Mapk1
Fc epsilon RI signaling pathway	1	Mapk1
Ferroptosis	1	Ftl1
FoxO signaling pathway	1	Mapk1
GABAergic synapse	1	Prkcb
Gastric cancer	1	Mapk1
Glucagon signaling pathway	1	Camk2a
Hippo signaling pathway	1	Ywhag
Human papillomavirus infection	1	Mapk1
Human T-cell leukemia virus 1 infection	1	Mapk1
IL-17 signaling pathway	1	Mapk1
Insulin resistance	1	Prkcb

Kaposi sarcoma-associated herpesvirus infection	1	Mapk1
Legionellosis	1	Rab1a
Malaria	1	Hbb-bs
Melanoma	1	Mapk1
NF-kappa B signaling pathway	1	Prkcb
NOD-like receptor signaling pathway	1	Mapk1
Olfactory transduction	1	Camk2a
Osteoclast differentiation	1	Mapk1
Pancreatic cancer	1	Mapk1
Parkinson disease	1	Camk2a
PD-L1 expression and PD-1 checkpoint pathway in cancer	1	Mapk1
Pertussis	1	Mapk1
Phosphatidylinositol signaling system	1	Prkcb
Phospholipase D signaling pathway	1	Mapk1
Platinum drug resistance	1	Mapk1
Prion disease	1	Mapk1
Progesterone-mediated oocyte maturation	1	Mapk1
Prolactin signaling pathway	1	Mapk1
Prostate cancer	1	Mapk1
Purine metabolism	1	Pde10a
Regulation of actin cytoskeleton	1	Mapk1
Relaxin signaling pathway	1	Mapk1
Renin-angiotensin system	1	Atp6ap2
Signaling pathways regulating pluripotency of stem cells	1	Mapk1
Spinocerebellar ataxia	1	Prkcb
Starch and sucrose metabolism	1	Pgm2l1
T cell receptor signaling pathway	1	Mapk1
TGF-beta signaling pathway	1	Mapk1
Th1 and Th2 cell differentiation	1	Mapk1
Th17 cell differentiation	1	Mapk1
Thyroid cancer	1	Mapk1
TNF signaling pathway	1	Mapk1
Toll-like receptor signaling pathway	1	Mapk1
Toxoplasmosis	1	Mapk1
Type II diabetes mellitus	1	Mapk1
Vasopressin-regulated water reabsorption	1	Dynll2

Yersinia infection	1	Mapk1

Supplementary Table 2. Biological pathways analysis of naïve R6/1 and trained R6/1 striatal EVs using the KEGG database of mice proteome. Frequency indicates the number of proteins significantly altered involved in a particular biological pathway.

**Biological pathway**	**Frequency**	**Protein ID**
Metabolic pathways	4	Pfkm; Atp6v1d; Pgam1; Atp5c1
Alzheimer disease	3	Mapk1; Psma7; Atp5c1
Central carbon metabolism in cancer	3	Pfkm; Mapk1; Pgam1
Chemical carcinogenesis	3	Mapk1; Mapk1; Atp5c1
Pathways of neurodegeneration	3	Mapk1; Psma7; Atp5c1
Prion disease	3	Mapk1; Psma7; Atp5c1
Amyotrophic lateral sclerosis	2	Psma7; Atp5c1
Biosynthesis of amino acids	2	Pfkm; Pgam1
Carbon metabolism	2	Pfkm; Pgam1
Glucagon signaling pathway	2	Pfkm; Pgam1
Glycolysis / Gluconeogenesis	2	Pfkm; Pgam1
HIF-1 signaling pathway	2	Pfkm; Mapk1
Human papillomavirus infection	2	Atp6v1d; Mapk1
Huntington disease	2	Psma7; Atp5c1
MicroRNAs in cancer	2	Mapk1; Fscn1
mTOR signaling pathway	2	Atp6v1d; Mapk1
Oxidative phosphorylation	2	Atp6v1d; Atp5c1
Parkinson disease	2	Psma7; Atp5c1
Synaptic vesicle cycle	2	Nsf; Atp6v1d
Thyroid hormone signaling pathway	2	Pfkm; Mapk1
Acute myeloid leukemia	1	Mapk1
Adherens junction	1	Mapk1
Adrenergic signaling in cardiomyocytes	1	Mapk1
African trypanosomiasis	1	Hbb-bs
AGE-RAGE signaling pathway in diabetic complications	1	Mapk1
Alcoholism	1	Mapk1
Aldosterone-regulated sodium reabsorption	1	Mapk1
AMPK signaling pathway	1	Pfkm
Apelin signaling pathway	1	Mapk1
Apoptosis	1	Mapk1
Autophagy	1	Mapk1

Axon guidance	1	Mapk1
B cell receptor signaling pathway	1	Mapk1
Bacterial invasion of epithelial cells	1	Septin3
Bladder cancer	1	Mapk1
Breast cancer	1	Mapk1
C-type lectin receptor signaling pathway	1	Mapk1
cAMP signaling pathway	1	Mapk1
Cellular senescence	1	Mapk1
cGMP-PKG signaling pathway	1	Mapk1
Chagas disease	1	Mapk1
Chemokine signaling pathway	1	Mapk1
Choline metabolism in cancer	1	Mapk1
Cholinergic synapse	1	Mapk1
Chronic myeloid leukemia	1	Mapk1
Circadian entrainment	1	Mapk1
Collecting duct acid secretion	1	Atp6v1d
Colorectal cancer	1	Mapk1
Coronavirus disease	1	Mapk1
Cushing syndrome	1	Mapk1
Diabetic cardiomyopathy	1	Atp5c1
EGFR tyrosine kinase inhibitor resistance	1	Mapk1
Endocrine resistance	1	Mapk1
Endometrial cancer	1	Mapk1
ErbB signaling pathway	1	Mapk1
Estrogen signaling pathway	1	Mapk1
Fc epsilon RI signaling pathway	1	Mapk1
Fc gamma R-mediated phagocytosis	1	Mapk1
Focal adhesion	1	Mapk1
FoxO signaling pathway	1	Mapk1
Fructose and mannose metabolism	1	Pfkm
GABAergic synapse	1	Nsf
Galactose metabolism	1	Pfkm
Gap junction	1	Mapk1
Gastric cancer	1	Mapk1
Glioma	1	Mapk1
Glutamatergic synapse	1	Mapk1
Glycine, serine, and threonine metabolism	1	Pgam1
GnRH secretion	1	Mapk1
GnRH signaling pathway	1	Mapk1

Growth hormone synthesis, secretion, and action	1	Mapk1
Hepatitis B	1	Mapk1
Hepatitis C	1	Mapk1
Hepatocellular carcinoma	1	Mapk1
Human cytomegalovirus infection	1	Mapk1
Human immunodeficiency virus 1 infection	1	Mapk1
Human T-cell leukemia virus 1 infection	1	Mapk1
IL-17 signaling pathway	1	Mapk1
Influenza A	1	Mapk1
Insulin signaling pathway	1	Mapk1
Kaposi sarcoma-associated herpesvirus infection	1	Mapk1
Leishmaniasis	1	Mapk1
Lipid and atherosclerosis	1	Mapk1
Long-term depression	1	Mapk1
Long-term potentiation	1	Mapk1
Malaria	1	Hbb-bs
MAPK signaling pathway	1	Mapk1
Melanogenesis	1	Mapk1
Melanoma	1	Mapk1
Natural killer cell mediated cytotoxicity	1	Mapk1
Neurotrophin signaling pathway	1	Mapk1
Neutrophil extracellular trap formation	1	Mapk1
NOD-like receptor signaling pathway	1	Mapk1
Non-small cell lung cancer	1	Mapk1
Oocyte meiosis	1	Mapk1
Osteoclast differentiation	1	Mapk1
Oxytocin signaling pathway	1	Mapk1
Pancreatic cancer	1	Mapk1
Parathyroid hormone synthesis, secretion, and action	1	Mapk1
Pathways in cancer	1	Mapk1
PD-L1 expression and PD-1 checkpoint pathway in cancer	1	Mapk1
Pentose phosphate pathway	1	Pfkm
Pertussis	1	Mapk1
Phagosome	1	Atp6v1d
Phospholipase D signaling pathway	1	Mapk1

PI3K-Akt signaling pathway	1	Mapk1
Platelet activation	1	Mapk1
Platinum drug resistance	1	Mapk1
Progesterone-mediated oocyte maturation	1	Mapk1
Prolactin signaling pathway	1	Mapk1
Prostate cancer	1	Mapk1
Proteasome	1	Psma7
Proteoglycans in cancer	1	Mapk1
Rap1 signaling pathway	1	Mapk1
Ras signaling pathway	1	Mapk1
Regulation of actin cytoskeleton	1	Mapk1
Relaxin signaling pathway	1	Mapk1
Renal cell carcinoma	1	Mapk1
Retrograde endocannabinoid signaling	1	Mapk1
Rheumatoid arthritis	1	Atp6v1d
RNA degradation	1	Pfkm
Salmonella infection	1	Mapk1
Serotonergic synapse	1	Mapk1
Signaling pathways regulating pluripotency of stem cells	1	Mapk1
Sphingolipid signaling pathway	1	Mapk1
Spinocerebellar ataxia	1	Psma7
T cell receptor signaling pathway	1	Mapk1
TGF-beta signaling pathway	1	Mapk1
Th1 and Th2 cell differentiation	1	Mapk1
Th17 cell differentiation	1	Mapk1
Thermogenesis	1	Atp5c1
Thyroid cancer	1	Mapk1
TNF signaling pathway	1	Mapk1
Toll-like receptor signaling pathway	1	Mapk1
Toxoplasmosis	1	Mapk1
Tuberculosis	1	Mapk1
Type II diabetes mellitus	1	Mapk1
Vascular smooth muscle contraction	1	Mapk1
Vasopressin-regulated water reabsorption	1	Nsf
VEGF signaling pathway	1	Mapk1
Viral carcinogenesis	1	Mapk1
Yersinia infection	1	Mapk1

Supplementary Table 3. Biological pathways analysis of naïve WT and trained WT striatal EVs using the KEEG database of mice proteome. Frequency indicates the number of proteins significantly altered involved in a particular biological pathway.

Biological pathways	Frequency	Protein ID
Alzheimer disease	1	Tubb5
Amyotrophic lateral sclerosis	1	Tubb5
Biosynthesis of amino acids	1	Pgk1
Carbon metabolism	1	Pgk1
Gap junction	1	Tubb5
Glycolysis / Gluconeogenesis	1	Pgk1
HIF-1 signaling pathway	1	Pgk1
Huntington disease	1	Tubb5
Insulin secretion	1	Snap25
Insulin signaling pathway	1	Prkar2b
Metabolic pathways	1	Pgk1
not_found	3	Sars1; Rgs8; Nipsnap2
Parkinson disease	1	Tubb5
Pathways of neurodegeneration	1	Tubb5
Phagosome	1	Tubb5
Prion disease	1	Tubb5
Salmonella infection	1	Tubb5
Synaptic vesicle cycle	1	Snap25

Supplementary Table 4. Biological pathways analysis of trained WT and R6/1 striatal EVs using the KEGG database of mice proteome. Frequency indicates the number of proteins significantly altered involved in a particular biological pathway.

Biological pathways	Frequency	Protein ID
Metabolic pathways	3	Pgk1; Pde1b; Pde10a
Parkinson disease	3	Tuba1b; Uba1; Uchl1
Pathways of neurodegeneration	3	Tuba1b; Uba1; Uchl1
Morphine addiction	2	Pde1b; Pde10a
Purine metabolism	2	Pde1b; Pde10a
African trypanosomiasis	1	Hbb-b2
Alzheimer disease	1	Tuba1b
Amyotrophic lateral sclerosis	1	Tuba1b
Apoptosis	1	Tuba1b
Biosynthesis of amino acids	1	Pgk1

Calcium signaling pathway	1	Pde1b
cAMP signaling pathway	1	Pde10a
Carbon metabolism	1	Pgk1
Gap junction	1	Tuba1b
Glycolysis / Gluconeogenesis	1	Pgk1
HIF-1 signaling pathway	1	Pgk1
Huntington disease	1	Tuba1b
Malaria	1	Hbb-b2
Olfactory transduction	1	Pde1b
Phagosome	1	Tuba1b
Prion disease	1	Tuba1b
Renin secretion	1	Pde1b
Salmonella infection	1	Tuba1b
Taste transduction	1	Pde1b
Tight junction	1	Tuba1b
Ubiquitin mediated proteolysis	1	Uba1
